# Role of Glutathione Depletion and Reactive Oxygen Species Generation on Caspase-3 Activation: A Study With the Kinase Inhibitor Staurosporine

**DOI:** 10.3389/fphys.2020.00998

**Published:** 2020-08-28

**Authors:** Aysenur Musaogullari, Alysia Mandato, Yuh-Cherng Chai

**Affiliations:** ^1^ Department of Chemistry, John Carroll University, University Heights, OH, United States; ^2^ Department of Chemistry, University of Pittsburgh, Pittsburgh, PA, United States

**Keywords:** staurosporine, caspase-3, apoptosis, reactive oxygen species, glutathione

## Abstract

Oxidative stress is known to contribute to the progression of apoptosis. Staurosporine is a broad-spectrum inducer of apoptosis, but its mechanism of action is not well understood. The goal of the present work was to elucidate the role of glutathione and reactive oxygen species (ROS) in the execution of staurosporine-induced apoptosis. HeLa cells were treated with staurosporine at 1 μM for up to 4 h. The concentration of glutathione, generation of ROS, and activation of caspase-3 were measured. The introduction of staurosporine significantly decreased the concentration of cellular glutathione and increased the presence of ROS after 3 h. These findings were concurrent with the activation of caspase-3. Interestingly, pre-treatment of cells with N-acetylcysteine, a precursor of glutathione, and a thiol antioxidant failed to block the depletion of glutathione, generation of ROS, and activation of caspase-3. Collectively, these results suggest that the cellular redox status may be one of the critical factors of the apoptotic pathway leading to caspase-3 activation by staurosporine.

## Introduction

Apoptosis is a highly regulated form of cell death during which the cell undergoes self-destruction (Kerr et al., 1972). Apoptosis occurs as a normal process in the maintenance of cell homeostasis, removing approximately the same number of cells that are generated each day. It also functions as a defense mechanism in immune reactions and in disease-related cell damage. Apoptosis must be highly regulated, as both excessive and insufficient cell death in the human body are linked to many types of cancer, autoimmune diseases, and neurodegeneration (Elmore, 2007; Singh et al., 2019). Therefore, it is of vital importance to potential disease therapies to clarify the factors that contribute to this form of programmed cell death.

It has been shown that apoptosis follows extrinsic or intrinsic pathways depending on the signal that initiates it (Elmore, 2007). Intrinsic apoptosis is consistently associated with alterations in redox status (Green and Reed, 1998; Coffey et al., 2000; Lv et al., 2019). The cellular redox potential is largely determined by glutathione, the most abundant, non-protein physiological antioxidant, and it exists in reduced (GSH) and oxidized (GSSG) forms. The tripeptide maintains the redox environment of the cell by the reduction of its cysteine residue to break disulfide bonds (Forman et al., 2009). Glutathione exists in both the cytosol and the mitochondria. Within the mitochondrion, coupled electron transport and oxidative phosphorylation produce reactive oxygen species (ROS), and the mitochondrial glutathione protects the organelle from oxidative stress caused by these events (Fernández-Checa et al., 1997; Armstrong et al., 2002). A decrease in the cellular antioxidant concentration is associated with the generation of an excess of ROS (Chae et al., 2000; Belmokhtar et al., 2001; Zhang et al., 2004; Omura et al., 2018).

**Graphical Abstract:**
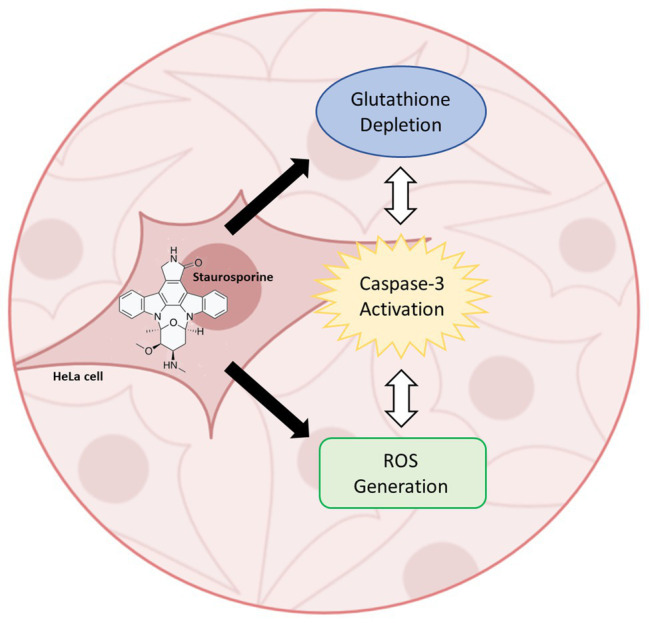
Staurosporine-induced activation of caspase-3 in HeLa cells involves the concurrent depletion of glutathione and generation of reactive oxygen species.

Previous studies have established that the direct treatment of cells with oxidants like hydrogen peroxide (Gardner et al., 1997) and diamide (Liu et al., 1999) induces apoptosis. Several non-oxidant apoptogenic agents such as tumor necrosis factor (Lee et al., 2002) and cycloheximide (Zhao et al., 2011) also elicit oxidative stress, indicating that ROS generation may be a conserved apoptotic event. However, the presence of an oxidative mechanism in apoptosis is unclear. While several studies suggest that ROS generation mediates the events leading to apoptosis (Ahlemeyer and Krieglstein, 1998; Kruman et al., 1998; Pong et al., 2001), other investigators suggest that antioxidant depletion, rather than ROS production, induces cell death (Ghibelli et al., 1995; Franco et al., 2007).

In addition to antioxidant depletion and ROS generation, apoptosis involves the activation of caspases. Caspases (cysteinyl aspartate proteases) are expressed as zymogens and become activated by an apoptotic signal. Once a caspase is activated, it may initiate an amplified apoptotic pathway by activating other caspases, leading to rapid cell death (Elmore, 2007). To date, 14 mammalian caspases have been identified and categorized into initiator, effector, and inflammatory caspases (Malsy et al., 2019; Singh et al., 2019). Caspase-3 is the major effector caspase activated by initiator caspases in the execution phase of apoptosis. Once activated, the protease cleaves proteins involved in DNA and cytoskeleton structure, leading to irreversible self-destruction (Elmore, 2007). The activation of caspase-3 may depend on redox status, but the optimal redox environment for activation is unclear (Hampton et al., 1998; Baker et al., 2000).

In addition to activation by initiator caspases, caspase-3 has been shown to be activated by staurosporine, a non-selective inhibitor of protein kinases (Chae et al., 2000; Omura et al., 2018; Malsy et al., 2019). Staurosporine is a well-known inducer of apoptosis, shown to activate endonucleases by a caspase-dependent mechanism (Belmokhtar et al., 2001; Zhang et al., 2004). However, the mechanism for the activation of caspase-3 by staurosporine is poorly understood. Staurosporine induction of apoptosis in colonic epithelial cells has been shown to be linked to intracellular glutathione efflux, with the efflux of glutathione accompanied by an increase in caspase-3 activity (Circu et al., 2009). In other studies, it has been shown that staurosporine-induced activation of apoptosis is mediated by ROS (Shimizu et al., 2004; Ramiro-Cortés et al., 2011). Although glutathione depletion and ROS generation have been independently shown in the mechanism of staurosporine-induced apoptosis, the relationship between the depletion of cellular glutathione, generation of ROS, and activation of caspase-3 is not known. In this work, we aim to understand the relationship between these events as they relate to oxidative stress.

## Materials and Methods

### Reagents

Staurosporine was purchased from MilliporeSigma (Billerica, MA). Dulbecco’s Modified Eagle’s Medium (DMEM), fetal bovine serum (FBS), 2',7'-dichlorodihydrofluorescein diacetate (DCFH-DA), tert-butyl hydrogen peroxide (t-BHP), N-acetylcysteine (NAC), dimethyl sulfoxide (DMSO), and metaphosphoric acid were all purchased from Sigma-Aldrich (St. Louis, MA). Cleaved caspase-3 monoclonal antibody, anti-rabbit IgG horseradish peroxidase (HRP)-linked antibody, and SignalFire Enhanced Chemiluminescent Reagents were purchased from Cell Signaling Technology (Danvers, MA). The GSH/GSSG Assay Kit was purchased from Aoxre Biosciences-Oxis Research (Portland, OR).

### Cell Culture

HeLa cells were obtained from the Lerner Research Institute of the Cleveland Clinic Foundation (Cleveland, OH). Cells were grown in medium supplemented with 10% FBS and were maintained in a humidified incubator at 37°C in 5% CO_2_ and 95% air. All experiments were conducted during the exponential growth phase of cells. Cells were pre-treated with 10 mM NAC in media for 12 h to supplement the cellular glutathione concentration. Apoptosis was induced by incubating cells with 1 μM staurosporine for the time indicated. Stock solutions of staurosporine and DCFH-DA were prepared in DMSO. For all treatments, the total DMSO concentration in media did not exceed 0.1%.

### Measurement of Cellular Glutathione

Cells were harvested by washing with and scraping into ice-cold phosphate-buffered saline (PBS). The cellular glutathione concentrations were measured enzymatically, as described previously by Tietze (1969) using the GSH/GSSG-412 kit. Briefly, samples were prepared in 5% metaphosphoric acid to remove proteins. Ellman’s reagent (5,5'-dithiobis-2-nitrobenzoic acid or DTNB), which reacts with reduced glutathione, was used to form a colored product spectrophotometrically detectable at 412 nm. Oxidized glutathione was recycled into reduced glutathione by glutathione reductase and β-nicotinamide adenine dinucleotide phosphate (NADPH). The rate of this reaction found by measuring the change in the absorbance was a linear function of the glutathione concentration in the reaction mixture. The parameters of a six-point calibration curve were used for glutathione concentration determination.

### Caspase-3 Cleavage Analysis by Western Blotting

Cells were scraped and lysed in buffer containing 10 mM Tris HCl (pH 7.5), 150 mM NaCl, 1% Nonidet P-40, 1 mM ethylenediaminetetraacetic acid (EDTA), and 1 mM ethylene glycol tetraacetic acid (EGTA). The cell lysates were then centrifuged at 14,000 *g* for 30 min. Samples were assayed in a spectrophotometer for protein concentration using the Bradford assay (Bradford, 1976) to normalize cell extracts to equal protein concentration. Proteins were separated by sodium dodecyl sulfate-polyacrylamide gel electrophoresis (SDS-PAGE) and transferred to polyvinylidene fluoride membranes. Membranes were blocked overnight in PBS containing 0.1% Tween-20 and 5% non-fat dry milk. Blots were incubated with cleaved caspase-3 antibody (1:1000) and with HRP-linked secondary antibody (1:1000) for 1 h. Blots were then visualized with enhanced chemiluminescence system.

### Determination of ROS Generation

ROS generation was measured by incubation with fluorescent probe DCFH-DA, as described previously by Hockenbery and co-workers (Hockenbery et al., 1993). t-BHP was used as a positive control for ROS since it is a well-known oxidant. The concentrations of staurosporine and t-BHP used were 1 and 200 μM, respectively. Around 10 μM DCFH-DA was added to the cell plates and incubated in the dark at 37°C in a humidified atmosphere (5% CO_2_ and 95% air). The incubation period with the dye solution was 1 h for all samples. When DCFH-DA enters cells, it is hydrolyzed by intracellular esterase to form DCFH, which is then oxidized to dichlorofluorescein (DCF) in the presence of ROS. The stained cells were washed once with ice-cold PBS, harvested, and resuspended in PBS supplemented with glucose. A PTI QuantaMaster dual-emission spectrofluorometer was used to analyze the production of DCF. The excitation and emission wavelengths were 500 and 525 nm, respectively.

### Statistical Analysis

The data are presented as mean ± standard error of the mean. All statistical differences of presented data were evaluated by one-way ANOVA followed by the *post hoc* least significant difference test. *p* < 0.05 was considered to be significant.

## Results

### Staurosporine Induces Caspase-3 Activation and Glutathione Depletion

**Figure 1:**
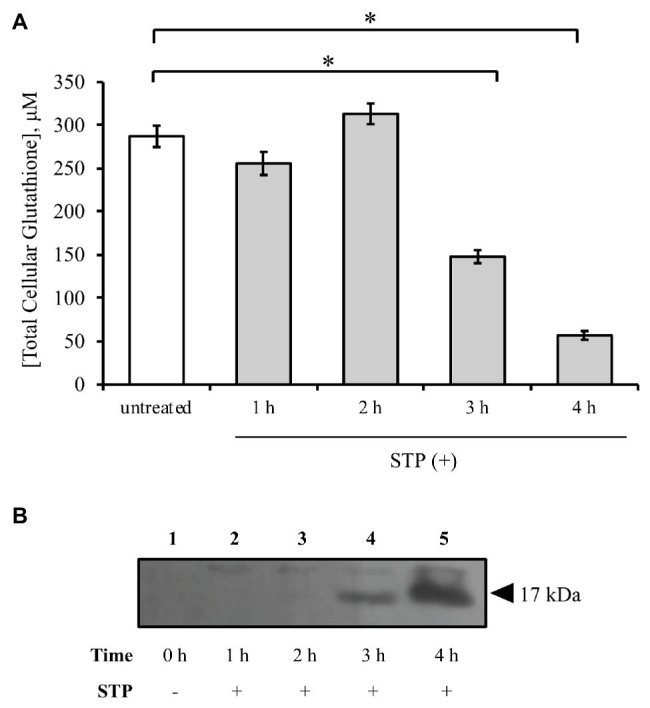
Staurosporine induces glutathione depletion concomitant with caspase-3 activation. **(A)** HeLa cells were exposed to 1 μM staurosporine for a 4-h time interval. The concentration of cellular glutathione was determined by an enzymatic recycling assay, as described under “Materials and Methods.” All results are expressed as mean ± standard error. **(B)** Western blot analysis of caspase-3 activation during a 4-h exposure to staurosporine. ^*^p < 0.05 against untreated control (*n* = 3).

The cellular glutathione concentration was measured at 1 h intervals for 4 h of treatment with 1 μM staurosporine. Over the same time-course, the cell lysates were analyzed on a western blot to determine the relationship between glutathione depletion and caspase-3 activation. Similar amounts of protein were measured between treatments (100 μg), indicating the absence of significant toxicity. Treatment of HeLa cells with staurosporine resulted in a time-dependent reduction of glutathione, first observed after 3 h (Figure 1A). Cleaved caspase-3, which has a molecular weight of approximately 17 kDa, was also first observed after 3 h (Figure 1B, lane 4). There was no glutathione loss and no activation of caspase-3 for up to 2 h of staurosporine treatment (Figure 1B, lanes 2 and 3). The kinetics of the activation of caspase-3 corresponds with the initiation of the staurosporine-induced depletion of glutathione. These results suggest that the depletion of glutathione may trigger the activation of caspase-3 by staurosporine.

### Staurosporine Induces Oxidative Stress by ROS Accumulation

**Figure 2:**
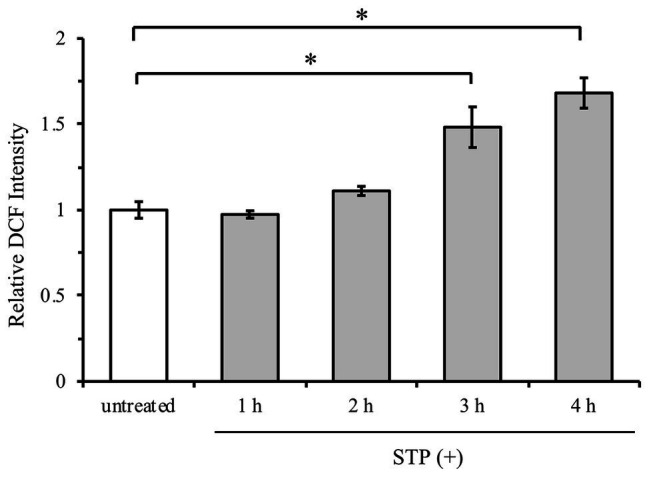
Staurosporine induces oxidative stress by reactive oxygen species (ROS) generation. HeLa cells were exposed to 1 μM staurosporine for a 4-h time interval. ROS generation was measured by incubation with fluorescent probe 2',7'-dichlorodihydrofluorescein diacetate (DCFH-DA), as described under “Materials and Methods.” Results are expressed as mean ± standard error. ^*^p < 0.05 against untreated control (*n* = 3).

To address whether staurosporine induces oxidative stress in HeLa cells, the occurrence of ROS was analyzed in a time-course experiment with staurosporine. ROS generation was measured using the fluorescent probe DCFH-DA, as explained under “Materials and Methods.” All ROS measurements are expressed relative to untreated cells. Staurosporine increased the intracellular ROS by 50% after 3 h and by 70% after 4 h of treatment (Figure 2). This increase in ROS generation at 3 h was concomitant with glutathione depletion (Figure 1A) and caspase-3 activation (Figure 1B).

### Glutathione Supplementation by NAC Does Not Block Caspase-3 Activation and ROS Generation

**Figure 3:**
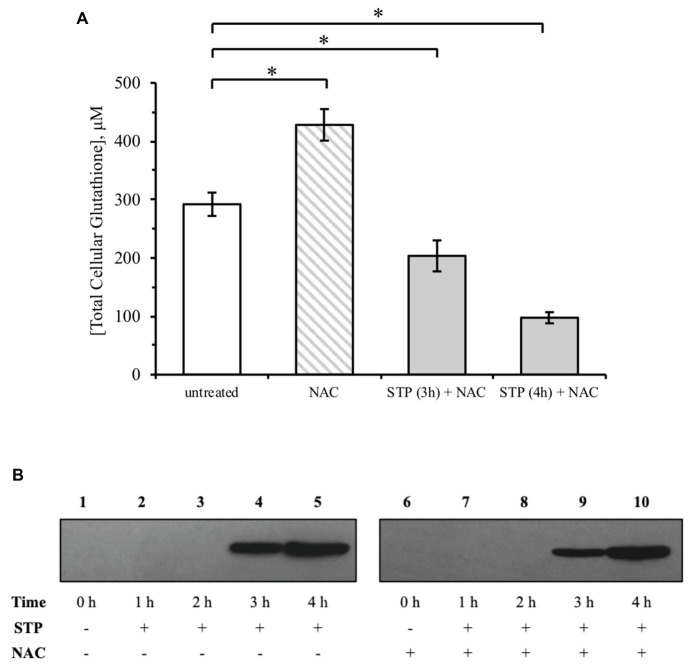
N-acetylcysteine (NAC) does not protect against staurosporine-induced glutathione depletion and caspase-3 activation. **(A)** HeLa cells were treated with 1 μM staurosporine without or with 12-h pre-treatment with 10 mM NAC. Glutathione concentration was determined by an enzymatic recycling assay, as described under “Materials and Methods.” Results are expressed as mean ± standard error. **(B)** Western blot analysis of caspase-3 activation by staurosporine with NAC pre-treatment. ^*^p < 0.05 against untreated control (*n* = 2).

Staurosporine treatment led to glutathione depletion and ROS generation, so these alterations in the cellular redox state may play a role in the regulation of caspase-3. To examine the effect of increasing the cellular glutathione concentration on caspase-3 activation, cells were pre-treated with 10 mM NAC for 12 h. Pre-treatment with NAC significantly increased the total glutathione concentration, but the depletion of glutathione by staurosporine continued (Figure 3A). Both untreated and NAC pre-treated cells had an approximately 50% decrease in cellular glutathione by 3 h and an 80% decrease by 4 h of treatment. Furthermore, NAC pre-treatment did not block the activation of caspase-3 (Figure 3B, lanes 9 and 10). These results suggest that an initial increase in cellular glutathione concentration is unable to alter the effects associated with staurosporine-induced caspase-3 activation.

**Figure 4:**
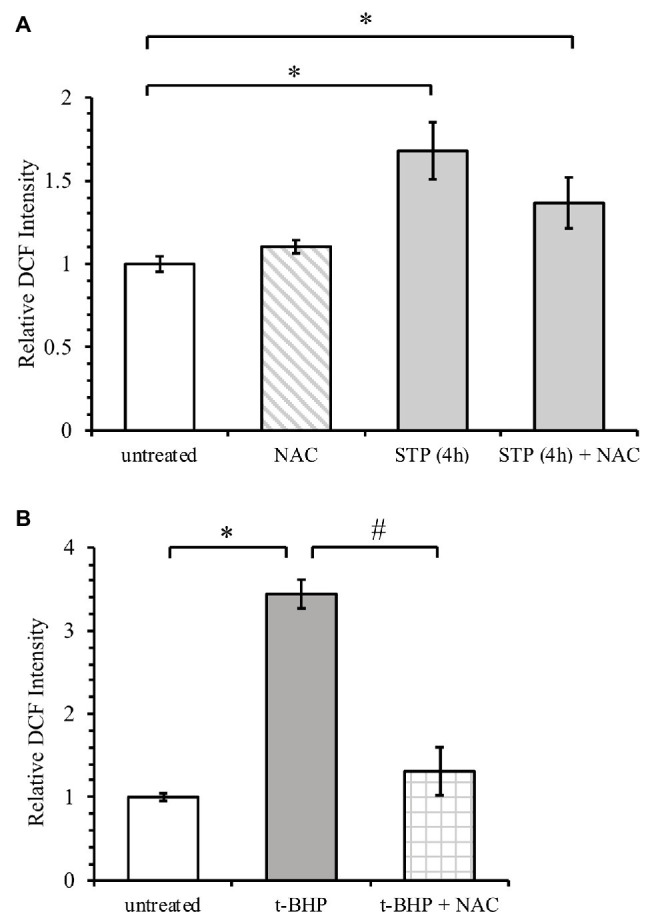
NAC attenuates ROS generation by tert-butyl hydrogen peroxide (t-BHP) but not by staurosporine. **(A)** HeLa cells were treated with 1 μM staurosporine without or with 12-h pre-treatment with 10 mM NAC. ROS generation was measured by incubation with fluorescent probe DCFH-DA, as described under “Materials and Methods.” **(B)** ROS generation after treatment of HeLa cells with 200 μM t-BHP. Results are expressed relative to untreated cells and presented as mean ± standard error. ^*^p < 0.05 against untreated control, ^#^*p* < 0.05 against t-BHP alone (*n* = 2).

An increase in glutathione may impact oxidative stress induced by staurosporine, so the effect of NAC pre-treatment on ROS generation was measured. t-BHP was used as a positive control for ROS because it mimics the ability of ROS to oxidize DCFH to fluorescent DCF. As shown in Figure 4, t-BHP increased ROS by over 3-fold relative to untreated cells. This effect was reversed with NAC pre-treatment. Regarding staurosporine, NAC pre-treatment had no significant effect on ROS generation (Figure 4).

## Discussion

Since its discovery in 1977, staurosporine has been studied extensively in the context of apoptosis. Although staurosporine is not clinically useful due to its lack of specificity, it serves as the parent compound for a variety of novel and highly successful anti-cancer drugs. One such example is imatinib, which is derived directly from staurosporine *via* a phenylaminopyrimidine derivative (Omura et al., 2018). Despite its popularity, the mechanisms underlying the effects of staurosporine are not well known. In the present work, we show that glutathione depletion, ROS generation, and caspase-3 activation are concurrent in the induction of apoptosis by staurosporine in HeLa cells (Graphical Abstract).

Glutathione is an important regulator of the cellular redox condition, and its depletion can disrupt various cellular processes. In this study, we show that staurosporine induces a significant decrease in cellular glutathione after 3 h of treatment. Multiple mechanisms are known to participate in the depletion of glutathione during apoptosis. In the presence of ROS, glutathione depletion is mainly attributed to its oxidation (Franco and Cidlowski, 2012). One study suggests that cytochrome c released from the mitochondria during apoptosis can catalyze S-nitrosoglutathione (GSNO) formation from GSH and nitric oxide (NO; Basu et al., 2010). More recent studies have focused on the role of glutathione extrusion by specific plasma membrane transporters as a significant contributor to apoptotic pathways (Franco and Cidlowski, 2012). Another contributor to glutathione depletion is protein S-glutathionylation, which refers to the formation of a protein-mixed disulfide between the thiol group of glutathione and a cysteine residue of a protein (Leonberg and Chai, 2007).

The depletion of glutathione and the generation of ROS upon staurosporine exposure have been separately evaluated in different preparations, including melanoma, neural, and epithelial cells (Shimizu et al., 2004; Zhang et al., 2004; Circu et al., 2009). For instance, Circu et al. show that the efflux of intracellular glutathione is accompanied by an increase in caspase-3 activity. However, the study suggests minimal ROS generation due to the absence of GSH oxidation. In contrast, our current observation shows that staurosporine induces a significant increase in ROS generation, concomitant with glutathione depletion and caspase-3 activation. It has previously been established that the ratio of GSH/GSSG remains very high, and GSH oxidation levels remain low inside the cytosol, even during conditions of oxidative stress (Forman et al., 2009). Thus, it is possible that staurosporine induces oxidative stress that does not result in GSH oxidation. Other studies have linked staurosporine exposure to ROS production, but in these studies, the involvement of glutathione depletion was not shown (Shimizu et al., 2004). To our knowledge, this is the first report to show the concurrence of glutathione depletion, ROS generation, and caspase-3 activation by staurosporine in a single study.

In cells, the availability of cysteine is the rate-limiting factor for glutathione *de novo* synthesis (Kerksick and Willoughby, 2005). Among the most widely used agents to increase the cellular cysteine pool is NAC. After uptake by the cell and deacetylation, NAC functions as a redox buffer and ROS scavenger (Erkkilä et al., 1998). A study by Park et al. shows that NAC inhibits ROS-dependent apoptosis in human conjunctival epithelial cells by regulating the mitochondria-dependent caspase activity (Park et al., 2015). Conversely, it has been shown that NAC may induce apoptosis through activation of the intrinsic mitochondrial signaling pathway (Liu et al., 2017). Here, we show that NAC alone did not induce the activation of caspase-3. Because caspase-3 activation is an early hallmark of cell death, this indicates that NAC alone does not induce apoptosis in HeLa cells under the given conditions.

Our results agree with earlier findings that NAC increases the total cellular glutathione concentration (Erkkilä et al., 1998). However, we show that NAC failed to protect against staurosporine-induced glutathione depletion and caspase-3 activation. Interestingly, NAC significantly inhibited ROS generation by t-BHP but not by staurosporine. One explanation for this result may be related to the identity of the ROS generated by treatment of cells with staurosporine as opposed to the ROS generated by a conventional potent oxidant. Mitochondrial ROS production in cells undergoing programmed cell death is complex, and it is known that the inhibition of ROS is specific to the antioxidant as well as the site of ROS generation (Liu and Schubert, 2009). Recent studies have shown that there are different pools of ROS with differing functions in the same cells (Cheung et al., 2016; Harris and DeNicola, 2020). Thus, the occurrence of staurosporine-induced apoptosis may require a particular oxidative environment, distinct from other apoptotic mechanisms.

Contrary to our observations, a previous study by Shimizu et al. shows that NAC almost completely reduces the ROS increase induced by staurosporine in HeLa cells (Shimizu et al., 2004). However, the reduction in ROS generation after NAC treatment is shown for a 30-min time course, where it is likely that caspase-3 may not have been activated. In contrast, our results show an increase in ROS after 3 h, concomitant with glutathione depletion and caspase-3 activation. The lack of effect of NAC treatment is likely due to the depletion of glutathione. Whether glutathione depletion results in ROS generation or vice versa is difficult to discern and requires further investigation. Interestingly, Shimizu et al. also show that NAC treatment results in a 60% reduction in staurosporine-induced caspase-3 activity. By contrast, our western blot results showed no effect of NAC treatment on the activation of caspase-3. It is possible that NAC may reduce the activity of caspase-3, but our observations indicate that NAC does not inhibit caspase-3 activation, which occurs by cleavage and is an irreversible process. Another possibility for the differences in results may be cell-type-specific or a function of staurosporine concentration and treatment period.

Although NAC failed to protect against staurosporine-induced glutathione depletion, ROS generation, and caspase-3 activation, the temporal relationship between these events emphasizes the vital role of an oxidative mechanism in apoptosis. Our findings and the literature suggest that the concurrent depletion of glutathione and generation of ROS may be required for the activation of caspase-3. Future work can provide further information on the types of ROS generated by staurosporine, as the fluorescent probe (DCFH-DA) used in this study may react with various types of ROS and oxidizing species and may be subject to other interfering reactions that could affect its signal (Kalyanaraman et al., 2012). The development and usage of more sensitive probes for ROS measurements will provide further insight into the specific identity and function of the ROS that mediate staurosporine-induced caspase-3 activation. This line of work will provide a better understanding of the biochemical mechanism of staurosporine, leading to the development of its novel derivatives as anti-cancer agents.

## Data Availability Statement

The raw data supporting the conclusions of this article will be made available by the authors, without undue reservation.

## Author Contributions

Y-CC conceived and designed the study. AMu and AMa performed the experiments. AMu and Y-CC analyzed the data and wrote the manuscript. AMa reviewed and edited the manuscript. All authors contributed to the article and approved the submitted version.

### Conflict of Interest

The authors declare that the research was conducted in the absence of any commercial or financial relationships that could be construed as a potential conflict of interest.
